# Healthy lifestyle behavior of employees in small and medium-sized enterprises in Aydin, Turkey

**DOI:** 10.12669/pjms.332.11757

**Published:** 2017

**Authors:** Safiye Ozvurmaz, Aliye Mandiracioglu

**Affiliations:** 1 Safiye Ozvurmaz, PhD. Department of Public Health Nursing, Nursing Faculty of Adnan Menders University, Aydin, Turkey; 2 Aliye Mandiracioglu, Professor of Department of Public Health, Ege University Faculty of Medicine, Izmir, Turkey

**Keywords:** Healthy lifestyle, Scale, Health promotion, Workplace

## Abstract

**Objective:**

To determine healthy lifestyle behavior and affecting risk factors in workers at small and medium-sized enterprises from four different sectors in Aydin, Turkey.

**Methods:**

This cross-sectional study was conducted at four different small and medium-sized enterprises in Aydin, Turkey and 264 employees participated in the study. A questionnaire was used for data collection. It consisted of questions about socio-demographic features (age, gender, marital status, education, perceived income, occupation and having children), health status, and medical history, medication use, having occupational accidents and occupational health and safety. Healthy Lifestyle Behavior Scale, which was developed by Walker et al. in 1996, was used to evaluate healthy lifestyle behaviors of the workers.

**Results:**

The mean score for Healthy Lifestyle Behavior Scale was 135.46±22.49. Gender, marital status, perceived income, sector of workplace, title, presence of a chronic disease, finding oneself healthy in the previous year and having an occupational accident in the previous year did not significantly affect any subscales of Healthy Lifestyle Behavior Scale. The workers aged over 50 years had significantly higher scores for health responsibility than those aged 20-29 years (p<0.05). The workers living in a village got significantly higher scores for Healthy Lifestyle Behavior Scale and its subscales health responsibility, physical activity, nutrition and spiritual development than those living in a city (p<0.05).

**Conclusion:**

Although workers have good spiritual development, they may not adopt physical activity as a healthy lifestyle and that workers benefiting from occupational health and safety services can display healthy lifestyle behavior.

## INTRODUCTION

Small and medium-sized enterprises (SMEs) greatly contribute to creation of job opportunities and high income; they are responsible for two-thirds of all jobs worldwide.^1^ SMEs comprise 99.8% of all enterprises and 74.2% of all employment in Turkey.^2^ Employees at SMEs have intensive workload and long working hours and work at high-risk work sites in most countries.^3^ Small and medium-sized workplaces have significant difficulty in managing health promotion and occupational health and safety worldwide.^4^ At SMEs in Turkey, insufficient health and safety measures are common in addition to job insecurity and unregistered work.

It is necessary to create a healthy working environment and to encourage employees to acquire health behavior. Differences in culture and social structure affect health behavior.^5^ Health promotion is a process which changes lifestyles and increases control of individuals over their health. Indicators of this process are health responsibility, physical activity, nutrition, spiritual development, interpersonal relationships and stress management.^6^ As well as work hours and psychosocial factors, physical and chemical risks lead to chronic illnesses and can become barriers to a healthy lifestyle.^7^ Workplaces are the most appropriate places for healthy lifestyle practices. A workplace directly influences physical, mental, economic and social wellbeing of workers and the time an individual spends there exceeds that spent in other locations. Group dynamics can easily be created among employees at a workplace as an organized and easily accessible community. In addition, health messages appropriate for special conditions of workplaces can be given.^4^ A healthy workplace and acquisition of a healthy lifestyle are the first prerequisite.^8^ At a workplace where a common policy for social support and healthy lifestyle has been adopted, employees with chronic diseases do not experience any difficulty.^9^ Health promotion activities include arrangement of organizational factors likely to affect health and support for a balance between work and life. Attempts to help employees to acquire a healthy lifestyle and to create a supportive environment should be undertaken together.^7^

Occupational disorders and accidents are the main problems in many countries. Nurses and other health staff at workplaces have to carry out a health protection and promotion program and to this aim they have to determine health behavior.^10^ It has been reported that occupational nurses spend most of their time on treatment related services and practices and do not have enough time for health promotion programs.^11^ It is necessary to know risk factors affecting health in order to protect and promote occupational health. It is important that occupational nurses and doctors should be able to describe healthy lifestyle behavior of employees and affecting factors. The aim of this study was to determine healthy lifestyle behavior and affecting risk factors in workers at SMEs from four different sectors in Aydin, Turkey.

## METHODS

This is a cross-sectional study. Convenience sampling was used and the study sample included readily available, four different SMEs from iron-steel, storage and delivery, shoe manufacturing and chestnut processing sectors in Aydin, Turkey, 2016. Access to all the employees of these SMEs (n=280 employees) was attempted. Ninety-four percent of 264 employees agreeing to participate in the study were contacted. Sixteen workers refused to have an interview because they were too busy. Data were collected at face to face interviews with a questionnaire. It consisted of questions about socio-demographic features (age, gender, marital status, education, perceived income, occupation and having children), health status, and medical history, medication use, having occupational accidents and occupational health and safety.

Data about healthy lifestyle behavior were gathered with Healthy Lifestyle Behavior Scale II (HLSBS II), which was developed by Walker et al. in 1996.^12^ The scale had been translated into Turkish and tested for its validity and reliability by Esin et al.^13^ The scale was composed of 52 items, and six factors; namely, health responsibility, physical activity, nutrition, spiritual development, interpersonal relationships and stress management. Cronbach alpha was reported to be 0.94 for the scale and ranged from 0.79 to 0.87 for its subscales.^12^ All items of the scale are affirmative statements. It is a four-point Likert scale ranging from 1 to 4 (1 corresponds to never, 2 sometimes, 3 usually and 4 regularly). Higher scores indicate positive healthy lifestyle behavior.^12^,^13^ This reliable and *valid Turkish scale was* used and all the participants completed it.

Written permissions were obtained from the workplaces where the study was conducted and ethical approval was taken from Tepecik Research and Training Hospital. All the participants gave oral informed consent.

Obtained data were analyzed with Statistical Package Program for Social Sciences 18.0. Mean values of obtained measures were presented together with their standard deviations and percentages. Independent samples t test and ANOVA were used to determine differences between groups. Statistical significance was set at p<0.005.

## RESULTS

Of 264 workers, 86.3% were male, 37.5% were primary school graduates, 63.4% were married, 31.8% were aged 30-39 years with a mean age of 35.22± 9.85 years and 44.2% had an income lower than their expenditures. Of all the workers, 29.5% were working in iron-steel manufacturing sector, 19.9% in store and distribution, 22.8% in chestnut processing and 27.8% in shoe manufacturing. Fifty-six point five percent of the participants noted that the place where they lived the longest was a city and 29.7% said they did not have any children.

Sixty-four point four percent of the workers did not have a regular physical examination, 65.5% found themselves healthy and 15.1% were using medications regularly. Twelve point five percent of the workers had a chronic disease and 71.2% were examined by an occupational health physician. Eighty-three point nine percent of the workers found workplace safety precautions sufficient and 12.4% had an occupational accident in the previous year.

Distribution of the scores by the subscales of HLSBS are shown in Table-III. The workers got the highest mean score for spiritual development and the lowest mean score for physical exercise.

**Table-I:** Distribution of the Workers by Certain Characteristics.

*Characteristics*	*N*	*%*
***Gender***
Male	228	86.3
Female	36	13.7
***Age (years)***
20<	10	3.7
20-29	74	27.7
30-39	85	31.8
40-49	60	22.5
50+	35	14.2
***Education***
Primary school	97	37.5
Secondary school	57	21.3
High school	63	23.6
University	47	17.6
***Marital status***
Single	93	34.8
Married	169	63.4
Divorced	2	1.8
***Perceived income***
Lower than expenditures	118	44.2
Equal to expenditures	111	41.6
Higher than expenditures	35	13.1
***Sector***
Iron-steel	76	29.5
Storage and distribution	53	19.9
Chestnut	61	22.8
Shoe making	74	27.8
***Status***
Blue collar	205	76.8
White collar	59	23.2
***Number of children***
None	79	29.7
1.00	52	19.5
2.00	98	37.7
3.00	26	9.7
4.00	9	3.4
***Place where the participants lived the longest***
Village	61	22.8
Small town	18	6.8
Town	37	13.9
City	148	56.5

**Table-II:** Health Status of the Workers participating in the Study

	*N*	*%*
***Having regular physicals activities***
Yes	95	35.6
No	169	64.4
***Perceived health status***
Yes	172	65.5
No	92	34.5
***Regular use of medications***
Yes	40	15.1
No	224	84.9
***Having a chronic disease***
Yes	33	12.5
No	231	87.5
***Does an occupational health physician check your health status regularly?***
Yes	187	71.2
No	77	28.8
***Do you find occupational safety precautions sufficient?***
Yes	220	83.3
No	44	16.7
***Having an occupational accident in the past one year***
Yes	30	12.4
No	234	87.6

**Table-III:** Scores for the Subscales of HLSBS

	*X ±SD*	*Minimum-maximum*
Health responsibility	21.78 ± 5.22	(10-34)
Physical activity	16.07 ± 5.44	(8-32)
Nutrition	21.65 ± 5.04	(11-33)
Spiritual development	28.17 ± 4.68	(14-36)
Interpersonal relationships	26.82 ± 4.05	(11-35)
Stress management	19.84 ± 4.64	(9-32)
Total score	135.46 ± 22.49	(77-200)

Gender, marital status, perceived income, sector of workplace, title, presence of a chronic disease, finding oneself healthy in the previous year and having an occupational accident in the previous year did not significantly affect any subscales of HLSBS. The workers aged over 50 years had significantly higher scores for health responsibility than those aged 20-29 years (p<0.05). The workers living in a village got significantly higher scores for HLSBS and its subscales health responsibility, physical activity, nutrition and spiritual development than those living in a city (p<0.05). (Table-IV)

**Table-IV:** The Distribution of the Scores for Healthy Lifestyle Behavior Scale and its Subscales of by Certain Socio-Demographic Features of the Workers

*Socio-Demographic Featuress*	*Health responsibility*	*Physical activity*	*Nutrition*	*Spiritual development*	*Interpersonal relationships*	*Stress management*	*Total*
***Gender***
Female	20.66±4.75	14.91±5.44	22.25±4.55	28.50+5.14	27.58+3.34	20.028+4.24	133.94+18.89
Male	22.30+5.11	15.99+5.03	22.40+4.92	28.34+4.30	27.14+4.04	20.09+4.83	136.86+22.37
t value	1.800	1.115	.162	2.578	-.614	0.75	.731
P	.073	.266	.871	.011	.539	.940	466
***Age Groups (years)***
20	21.22±4.43	14.66±4.79	22.88±3.72	29.55±4.95	27.70±3.56	18.77±3.92	134.88±21.0
20-29	20.71±4.86	16.12±6.17	20.81±5.27	27.86±4.82	26.75±3.60	19.61±4.96	132.51±23.9
30-39	21.85±5.11	15.58±5.21	22.73±4.77	28.29±4.56	26.73±4.06	19.59±4.90	134.98±21.8
40-49	23.49±4.68	15.91±4.70	23.23±4.17	28.81±4.12	27.88±4.24	20.70±4.10	141.05±18.4
50+	23.31±5.64	16.08±5.52	23.31±4.96	28.57±3.52	28.00±3.90	21.51±4.96	140.80±22.3
F value	3.189	0.223	2.921	0.567	1.363	1.630	1.672
P	0.014	0.926	0.222	0.687	0.247	0.167	0.157
***Education***
Primary school	23.40±4.98	15.77±4.73	23.42±4.50	28.90±4.15	27.66±3.99	21.36±4.20	140.80±19.3
Secondary school	22.28±4.47	16.19±5.92	22.37±4.74	28.81±4.11	27.45 ±3.98	19.76±5.18	138.52±21.9
High school	20.61±5.67	15.06±5.76	21.38±5.19	27.11±4.82	26.25±4.26	18.85±5.15	129.31±24.3
University	21.06±4.55	16.61±5.51	21.57±4.98	28.39±4.55	27.25±3.24	19.45±4.22	134.71±21.4
F value	4.682	0.837	2.771	2.331	1.363	4.205	4.661
P	0.003	0.9475	0.042	0.075	0.247	0.006	0.004
***Marital status***
Married	21.45±5.27	16.40±6.16	21.75±5.21	28.45±5.15	27.00±4.27	20.33±5.07	136.31±24.9
Single	22.39±4.90	15.48±4.86	22.66±4.62	28.36±3.90	27.31±3.78	19.95±4.56	136.43±19.8
Divorced	23.50±12.0	21.00±9.89	27.50±4.94	25.00±9.89	27.50±3.53	20.00±8.48	144.50±48.7
F value	1.074	1.792	2.141	.595	.199	.187	.136
P	0.343	0.169	0.120	0.552	0.820	0.829	0.873
***The place where the workers lived the longest***
Village	22.83±5.05	16.61±4.53	22.94±4.21	29.28±3.47	27.62±3.73	20.59±4.37	140.92±15.5
Small town	21.88±4.83	15.05±6.15	21.25±5.34	26.82±4.44	25.94±4.50	19.88±4.75	131.12±24.1
Town	19.83±3.91	13.64±4.44	20.16±4.13	25.83±4.21	26.05±3.43	18.59±3.74	124.16±16.7
City	22.34±5.27	16.18±5.72	22.82±5.08	28.81±4.55	27.47±4.04	20.27±5.08	138.40±23.7
F value	2.977	2.810	3.542	6.329	2.101	1.539	5.517
P	0.032	0.040	0.015	0.000	0.101	0.205	0.001
***Perceived income***
Lower than expenditures	21.97±4.88	16.38±5.42	22.30±4.89	20.63+2.72	26.94±3.99	19.57+4.62	135.67±21.5
Equal to expenditures	22.02±5.43	15.28±5.23	22.23±4.78	21.07±5.01	27.53±3.69	20.63+4.78	136.51±21.6
Higher than expenditures	22.58±4.71	15.82±5.76	23.12±5.10	22.56±6.12	27.08±4.59	20.09+5.05	139.03±24.41
F value	0.199	1.184	0.441	0.895	0.264	1.405	0.292
P	0.819	0.308	0.644	1.025	1.24	0.247	0.747
***Sector***
Iron-steel manufacturing	23.40±4.98	15.83±5.68	22.41±5.12	28.18±4.89	26.90±4.52	20.38±4.91	137.85±23.6
Storage and distribution	22.28±4.47	16.20±5.28	22.28±4.65	27.92±4.89	26.48±3.83	19.34±4.51	133.18±21.3
Chestnut processing	20.61±5.67	16.06±5.38	22.73±4.95	28.65±4.29	27.29±3.61	19.90±4.58	136.86±21.6
Shoe manufacturing	21.06±4.55	15.41±5.26	22.13±4.76	28.62±3.68	27.93±3.60	20.45±4.91	136.91±21.0
F value	0.654	0.125	0.174	0.379	1.55	0.696	0.467
P	0.581	0.746	0.914	0.768	0.200	0.555	0.706
***Status***
Blue collared	21.97±5.01	15.53±5.44	22.12±4.94	28.37±4.25	27.21±3.93	19.98±4.75	135.81±21.8
White collared	22.42±5.34	16.91±5.11	23.25±4.51	28.35±4.96	27.16±4.04	20.44±4.77	138.55±22.0
t value	-.595	-1.734	-1.574	0.027	0.082	-.654	-.842
P	0.552	0.084	0.117	0.978	0.934	0.514	0.401
***Having regular physicals activities***
Yes	23.63±5.32	17.01±5.54	23.44±4.75	28.73±4.23	27.40±3.76	21.43±4.66	142.09±22.6
No	21.22±4.75	15.20±5.21	21.80±4.84	28.16±4.51	27.10±4.06	19.34±4.65	133.43±20.94
t value	3.734	2.618	2.600	0.985	0.588	3.460	3.039
P	0.000	0.009	0.010	0.326	0.557	0.001	0.003
***Finding oneself healthy in the previous year***
Yes	22.36±5.14	16.28±5.57	22.81±4.87	28.61±4.55	27.12±4.14	20.32±4.73	138.32±22.21
No	21.54±4.95	15.04±4.98	21.59±4.76	27.91±4.12	27.35±3.59	19.65±4.78	133.03±21.02
t value	1.228	1.781	1.932	1.215	-.432	1.086	1.871
P	0.221	0.076	0.054	0.226	0.666	0.279	0.063
***Having an occupational accident in the previous year***
Yes	20.96±3.28	14.51±5.25	21.68±4.69	28.35±3.23	27.96±2.65	18.96±3.94	132.60±15.2
No	22.19±5.25	16.04±5.38	22.44±4.88	28.39±4.54	27.09±4.08	20.24±4.83	136.94±22.6
t value	-1.225	-1.446	-.807	-.049	1.551	-1.366	-.985
P	0.222	0.149	0.425	0.961	0.128	0.173	0.326
***Regular use of medications***
Yes	23.84±4.25	14.74±4.11	23.44±4.43	29.12±5.02	27.52±4.16	21.46±3.87	141.15±18.43
No	21.77±5.16	16.04±5.57	22.19±4.92	28.23±4.29	27.14±3.92	19.84±4.85	135.62±22.40
t value	2.335	-1.387	1.466	1.165	0.552	1.973	1.438
P	0.020	0.167	0.144	0.245	0.581	0.050	0.152
***Having a chronic disease***
Yes	22.40±4.61	14.96±4.68	22.34±4.45	28.34±4.57	26.78±4.17	20.54±3.61	135.81±20.1
No	22.03±5.15	15.97±5.48	22.38±4.93	28.37±4.40	27.26±3.92	20.01±4.89	136.55±22.2
t value	0.425	-1.125	-.050	-.034	-.622	0.745	-.191
P	0.673	0.267	0.960	0.973	0.538	0.460	0.849
***Having a regular physical examination by an occupational health physician***
Yes	22.85±4.83	15.86±4.99	23.21±4.60	28.75±3.98	27.71±3.70	20.63±4.43	139.43±19.1
No	20.19±5.21	15.80±6.28	20.31±4.89	27.40±5.25	26.00±4.28	18.75±5.23	129.00±26.37
t value	3.934	0.081	4.497	2.242	3.248	2.953	3.465
P	0.000	:0.935	:0.000	0.026	0.001	0.003	0.026
***Finding workplace safety precautions sufficient***
Yes	22.42±4.94	15.87±5.21	22.75±4.77	28.64±4.16	27.29±3.80	20.30±4.56	137.77±20.4
No	20.19±5.53	15.65±6.34	20.41±4.93	26.92±5.46	26.83±4.71	18.95±5.52	129.45±27.7
t value	2.601	0.249	2.862	2.281	0.685	1.714	1.851
P	0.010	0.804	0.005	0.023	0.494	0.088	0.002
***Compliance with workplace safety measures***
Yes	22.37±5.04	16.06±5.44	22.72±4.88	28.64±4.34	27.43±3.82	20.20±4.76	137.96±21.5
No	19.60±4.92	14.00±4.80	19.60±3.71	26.21±4.58	25.44±4.59	19.13±4.62	124.35±21.4
t value	2.745	1.954	3.255	2.779	2.578	1.138	3.154
P	0.006	0.050	0.001	0.006	0.011	0.256	0.020

There was a significant difference in the scores for the scale in terms of education (F: 4.661, p: 0.004). The primary school graduates got the highest scores. The workers having a regular physical examination by an occupational health physician (t: 2.242, p: 0.026), those finding workplace safety measures sufficient (t: 1.851, p: 0.002) and those reporting to comply with workplace safety measures (t: 3.154, p: 0.020) got significantly higher scores for the scale. (Table-IV)

## DISCUSSION

In the present study, healthy lifestyle behavior of workers at SMEs from four different sectors of work was determined. Prior relevant studies included workers from only one sector of work. There have not been any studies on SMEs. The mean score for HLSBS was 135.46±22.49. Using the same scale, many researchers reported lower scores in Turkish workers.^13^-^17^ This study was conducted in a well-developed region close to Aegean Sea. Therefore, the workers had healthier lifestyle behavior although they worked at SMEs.

In the current study, the workers had the highest score for spiritual development followed by interpersonal relationships, health responsibility, nutrition, stress management and physical activity, which is consistent with the findings reported by Küçük.^16^ In other words, spiritual development is the healthy lifestyle behavior most contributing to health promotion among the workers.^16^,^18^ The finding of high scores for spiritual development can be attributed to culture and place of living. Küçük suggests that spirituality is experiences unlikely to be acquired through five senses but transcendence by means of inner peace, harmony or connectedness to others.^16^,^18^ The workers had high scores for interpersonal relationships. It may be that they were an organized community, spent most of their time at work and had positive relationships with their colleagues, families and relatives. Social support contributes to acquisition of health behavior and protection against risks at workplaces.^19^

It has been reported that workers usually have poor physical activity. It can be suggested that long working hours and poor working conditions have a negative impact on healthy lifestyle behavior.^20^

The workers did not differ in their scores for nutrition and health responsibility. In the region where the study was performed, people usually have a Mediterranean diet and consume high amounts of olive oil, fruit and vegetables. Lack of sufficient care and attention to nutrition can be due to heavy workload and poor physical conditions of workplaces may cause workers to skip meals and follow an insufficient, unbalanced and unhealthy diet. Pappas et al.^21^ from the United States found that bus drivers had unhealthy eating habits.

Stress management involves to what extent individuals know sources of stress and use stress control mechanisms. In the present study, the workers did not get high scores for stress management. It may be due to problems encountered at workplaces, heavy workload, working hours, time constraints and low incomes. It is important for occupational nurses who design occupational health promotion programs to be able to describe healthy lifestyle behavior and recognize affecting factors for stress management at workplaces.^11^

Gender, marital status, perceived income, work sector, presence of a chronic disease, considering oneself healthy in the previous year and having an occupational accident in the previous year did not significantly affect any of the subscales of HLSBS. This finding is compatible with results of studies on workers from the textile sector and various other industrial areas.^14^,^15^

The primary school graduates got the highest score for healthy lifestyle behavior, which is congruent with the results of prior studies from Turkey.^15^ It may be that workers with low education levels pay more attention to recommendations about healthy lifestyle behavior by occupational health professionals.

The workers who had regular physicals by occupational health physicians and those considering occupational safety measures sufficient got higher scores for healthy lifestyle behavior. This emphasizes that regular check-ups by health staff at workplaces can prevent many health problems and help workers acquire a healthy lifestyle.

There has been a debate about health promotion at SMEs recently. Occupational health nurses and physicians are responsible for health promotion at these enterprises. They contribute to acquisition of health behavior ad creation of a safe and healthy working environment.

In the present study, the workers got low scores for physical activity, but high scores for spiritual development. The workers reporting to benefit from occupational health and safety services received higher scores for healthy lifestyle behavior. It can be recommended that importance should be placed on counseling for sufficient, balanced and healthy nutrition and physical activity at workplaces and raising awareness for exercise and nutrition. In addition, occupational health and safety services should be supported and offered at all SMEs.
